# Nutrition and Cancer—From Prevention to After-Care

**DOI:** 10.3390/nu18172926

**Published:** 2026-09-07

**Authors:** Zhenhua Liu

**Affiliations:** 1 Nutrition and Cancer Prevention Laboratory, School of Public Health and Health Sciences, University of Massachusetts, Amherst, MA 01003, USA; zliu@nutrition.umass.edu; Tel.: +1-413-545-1075; 2 UMass Cancer Center, University of Massachusetts Chan Medical School, Worcester, MA 01605, USA

Cancer is a major contributor to global disease burden in the 21st century, accounting for nearly one in six deaths (16.8%) and almost one in four deaths (22.8%) attributable to noncommunicable diseases worldwide [1,2]. Globally, excluding non-melanoma skin cancers, the number of new cancer cases has more than doubled since 1990, reaching 18.5 million in 2023, while cancer-related deaths have risen by 74%, totaling 10.4 million [3]. In 2026, approximately 2,114,850 new cancer cases and 626,140 cancer deaths are projected to occur in the United States [4]. The global number of new cancer cases is projected to increase by 61% over the next 25 years, reaching 30.5 million by 2050, while annual cancer-related deaths are expected to rise by nearly 75%, climbing to 18.6 million [5].

While the overall number of cancer cases and deaths is set to rise substantially from 2024 to 2050, the increasing rates in older adults (age > 50 years) have generally remained stable or declined. However, cancers diagnosed in adults under 50 (defined as early-onset cancer) have risen significantly over recent decades, particularly for breast, colorectal, and uterine tumors [6,7,8,9]. The rapidly increasing incidence of early-onset cancer highlights the urgent need to elucidate the contribution of emerging risk factors and to develop novel strategies for its prevention and interception [6,10,11,12].

Alongside the significant rise in new cancer cases among younger individuals, the number of people living with a history of cancer continues to grow; this increase is driven by population growth and aging, as well as advancements in early detection, treatment and supportive care that have improved survival rates. There are close to 54 million people worldwide who were diagnosed with cancer diagnosis within the last five years [13]. As of 1 January 2025, approximately 18.6 million people in the United States were living with a history of cancer, and this figure is expected to surpass 22 million by 2035 [14,15].

With the continuous increase in new cancer cases, particularly the rising incidence of cancer in young adults (<50 years), and the growing number of cancer survivors, the Special Issue “Nutrition and Cancer—From Prevention to After-Care” in *Nutrients* brings together six contributions, spanning mechanistic cell studies, animal models, clinical pilot trials, behavioral intervention work, and systems-level modeling, that focus on dietary strategies from cancer prevention to after-care. These publications converge on an important message: nutrition-related exposures—dietary patterns, bioactive compounds, microbiome-modulating substrates, and supplements—interact with metabolic and signaling pathways in ways that may be clinically meaningful, but only when evaluated with appropriate attention to biological context and translational feasibility.

**Mechanistic Foundations: Nutrition-Related Molecules and Tumor-Selective Effects**

A cornerstone of nutrition-oncology research is establishing mechanistic plausibility while addressing a recurring translational challenge: interventions should ideally inhibit malignant phenotypes while sparing—or even supporting—normal tissues. Zeng et al. (Contribution 1) address this concept through the study of ursodeoxycholic acid (UDCA), a hydrophilic bile acid with anti-inflammatory properties that may be influenced by dietary patterns and that has been implicated in attenuating aspects of colon carcinogenesis. Importantly, their work demonstrates that UDCA exhibits greater inhibitory effects on proliferation in cancerous HCT116 colon cells compared with noncancerous NCM460 colon cells. This tumor-selective pattern strengthens the rationale for further mechanistic and translational investigation of bile-acid-related pathways as mediators linking diet to colorectal cancer risk and progression. More broadly, the study highlights the value of comparative testing across malignant and nonmalignant models when evaluating dietary bioactive components.

Centeno et al. (Contribution 2) examine a complementary but distinct clinical concern: whether a commonly used nutritional supplement could modulate chemotherapy-related cellular responses in ways that are protective for normal tissues yet potentially problematic in tumor cells. Taurine, a non-proteogenic amino acid frequently used as a supplement, has been reported to protect tissues from degeneration in contexts involving DNA-damaging agents such as cisplatin. Using modeling of intracellular taurine levels associated with ovarian cancer, the authors identify activation of pathways linked to cell protection and stress responses, including p53, ERK, mTOR, and DNA-damage-sensing-dependent mechanisms. This contribution underscores a critical principle for supportive nutrition in oncology: interventions intended to reduce toxicity must be evaluated not only for symptom benefits but also for plausible effects on tumor cell survival pathways, particularly in the setting of DNA-damaging therapy. Such work supports the development of more nuanced guidance that is sensitive to tumor biology, treatment modality, and timing of supplementation.

**Life-Course Prevention and the Microbiome: Early Exposures with Long-Term Consequences**

The rising incidence of young-onset colorectal cancer has sharpened attention on early-life determinants of later cancer risk. Lin et al. (Contribution 3) contribute to this area by demonstrating that *Antrodia camphorata* supplementation during early life alters gut microbiota and inhibits young-onset intestinal tumorigenesis later in life in APC1638N mice. The study aligns with an emerging life-course model in which early nutritional exposures shape microbial ecology and host physiology during developmental windows that may influence long-term susceptibility to tumorigenesis.

From a translational perspective, this work is notable for integrating prevention with microbiome biology and for pointing toward potential mechanistic intermediates beyond dietary intake itself, including microbiota composition and downstream microbial metabolites. At the same time, it reinforces the need for careful stepwise translation—particularly regarding dosing, safety, durability of effects, and generalizability to diverse human populations—before early-life supplementation approaches can be responsibly advanced.

**Metabolic and Microbiome-Targeted Strategies in Pancreatic Cancer: Promise, Safety, and Pragmatism**

Pancreatic ductal adenocarcinoma (PDAC) remains a leading cause of cancer mortality, prompting interest in adjunctive strategies that target tumor metabolism or treatment responsiveness. Two publications in this Special Issue address PDAC-relevant nutrition interventions from distinct angles.

Cortez et al. (Contribution 4) investigate the impact of a ketogenic diet (KD) on late-stage pancreatic carcinogenesis in mice, explicitly evaluating both efficacy and safety. The KD has been proposed to exert anti-tumor effects via altered systemic and tumor metabolism, yet concerns persist regarding tolerability, adverse metabolic consequences, and heterogeneity in tumor metabolic dependencies. By examining late-stage carcinogenesis and reporting on safety considerations alongside efficacy signals, this study reflects an important maturation of the field: diet-pattern interventions in oncology must be evaluated with clinical realism, including the potential constraints of advanced disease physiology and the practical requirements for implementation.

In a complementary clinical pilot, Nakaoka et al. (Contribution 5) assessed the efficacy of 1-kestose supplementation in patients with PDAC in a randomized controlled design. Motivated by evidence that the gut microbiota may influence chemotherapy response, the authors explore whether a fructooligosaccharide prebiotic can provide clinical benefit during chemotherapy. This contribution is particularly valuable as an interventional step beyond association studies, advancing the microbiome from a descriptive biomarker space toward a modifiable therapeutic target. It also exemplifies the methodological challenges that define nutrition trials in oncology—patient heterogeneity, concurrent supportive care, adherence variability, and endpoint selection—while nevertheless demonstrating the feasibility and relevance of microbiome-oriented strategies in a high-need clinical setting.

**Survivorship and Late Effects: Translating Evidence into Actionable Nutrition Support**

As survivorship grows, late effects of treatment increasingly define long-term well-being. Pritlove et al. (Contribution 6) address a persistent clinical gap: gastrointestinal toxicity following pelvic radiotherapy in gynecologic cancer survivors. Their pilot study evaluates an innovative culinary nutrition intervention (EDIBLE) designed to reduce digestive irregularities and improve dietary decision-making in a context where standard guidance is often nonspecific and difficult to operationalize.

This contribution underscores that effective nutrition care is not solely a matter of identifying “what to eat,” but also “how to make it feasible”—through skills-based education, symptom-informed adaptation, and behavioral support. It further highlights the importance of endpoints centered on patient experience, including symptom burden and quality of life, which frequently determine adherence and real-world impact even when mechanistic rationale is strong.

Taken together, the six publications emphasize several priorities for the next phase of nutrition-and-cancer research. First, context specificity is essential: nutrition-related compounds may exert differential effects across malignant and nonmalignant tissues and may interact with therapy-specific mechanisms, reinforcing the need for comparative models and treatment-interaction studies. Second, the microbiome, metabolism, and signaling are increasingly inseparable domains; future work will benefit from integrated approaches combining microbiome profiling, metabolomics, and pathway-level analyses. Third, translation requires balanced attention to efficacy and safety, particularly in advanced cancers and during active therapy. Finally, implementation-oriented interventions—such as culinary nutrition programs—should be expanded and rigorously evaluated, as they represent a practical pathway to deliver evidence-based support in survivorship and beyond.

In conclusion, this collection highlights nutrition as a biologically active, clinically relevant, and increasingly precision-oriented component of cancer prevention, treatment support, and after-care. By spanning tumor-selective bioactive effects (UDCA), pathway-aware modeling of supplementation in the context of chemotherapy (taurine), early-life microbiome modulation with long-horizon prevention implications (*Antrodia camphorata*), metabolic dietary pattern testing with explicit safety considerations (ketogenic diet), microbiome-targeted clinical intervention (1-kestose), and actionable survivorship nutrition support (EDIBLE), these contributions collectively advance the field toward more rigorous and patient-centered nutrition-oncology frameworks.

## Data Availability

No new data were created or analyzed in this study. Data sharing is not applicable to this article.
